# Clinical and functional characteristics of the *KLF11* (p.R29Q) variant associated with MODY7

**DOI:** 10.3389/fendo.2026.1781016

**Published:** 2026-03-11

**Authors:** Yidan Qiao, Yanyan Jiang, Mengyang Zhang, Yi Song, Dan Song, Ying Xin, Guijun Qin, Yanxia Liu

**Affiliations:** 1 Department of Endocrinology and Metabolism, The First Affiliated Hospital of Zhengzhou University, Zhengzhou, China; 2 Department of Geriatric Endocrinology and Metabolism, The First Affiliated Hospital of Zhengzhou University, Zhengzhou, China

**Keywords:** diabetes, functional studies, gene mutation, KLF11, MODY

## Abstract

**Objective:**

The *KLF11* gene can cause maturity-onset diabetes of the young type 7 (MODY7). Currently, there are few reports on MODY7 cases and their clinical and functional characteristics. This study aimed to analyze the clinical and functional features of the *KLF11* R29Q variant to provide a basis for the diagnosis and treatment of this disease.

**Methods:**

Clinical baseline data of patients were collected, and high−throughput sequencing (NGS) was used to screen probands clinically suspected of harboring *KLF11* variants, with validation performed in their family members. A luciferase reporter assay was used to test whether the *KLF11* R29Q variant binds to the insulin promoter. Real-time PCR, western blotting, and glucose-stimulated insulin secretion (GSIS) assays were employed to analyze insulin expression and insulin secretion activity in β-cell lines.

**Results:**

1) Clinical data showed that Proband 1 presented with insulin dependence, severe hyperlipidemia, diabetic retinopathy, and cataract at the initial stage of the disease. A relatively high dose of insulin was required and could not be completely discontinued; after insulin treatment, glucose and lipid metabolic disorders and visual acuity were improved. Proband 2 exhibited poor islet function at early onset, accompanied by diabetic autonomic neuropathy and peripheral neuropathy, and glycemic control remained unsatisfactory despite insulin therapy. 2) Genetic testing demonstrated that the heterozygous *KLF11* R29Q variant was identified in Proband 1, her father, and her elder sister, as well as in Proband 2 and her mother. The elder sister of Proband 1 was in a prediabetic state, whereas this variant was not detected in Proband 1’s mother or younger brother. The mother of Proband 2 had normal blood glucose levels and displayed no diabetes-related clinical manifestations. 3) Protein three-dimensional structure predictions indicated that the *KLF11* R29Q mutation affects the electrostatic potential on the surface of the *KLF11* protein molecule, disrupting its tertiary structure and compromising protein stability. 4) Cell-based luciferase reporter assays using wild-type and mutant constructs demonstrated that the *KLF11* R29Q variant had impaired insulin promoter regulatory activity. Furthermore, real-time PCR, western blotting, and GSIS analyses indicated that this variant impairs insulin expression in pancreatic β-cells and inhibits insulin secretion.

**Conclusion:**

We studied a *KLF11* R29Q single-gene mutation associated with MODY7. The study confirmed that this variant has impaired insulin promoter regulatory activity and inhibits insulin expression and secretion.

## 1 Introduction

Maturity-onset diabetes of the young (MODY) is a type of autosomal dominant genetic disorder caused by single-gene mutations leading to defective pancreatic β-cell function. It accounts for approximately 1% to 5% of all diabetes cases, typically presenting before the age of 35, and more likely before 25 (1). A total of 14 subtypes of MODY have been reported to date. MODY7, caused by mutations in the *KLF11* gene, is particularly rare. Different subtypes of MODY display marked heterogeneity with respect to age at onset, severity of pancreatic β-cell dysfunction, insulin dependence, and risk of complications, clinical diagnosis is mainly based on a comprehensive evaluation of family history, clinical phenotypic characteristics, and molecular genetic testing results. However, due to the overlapping clinical manifestations between some patients and type 1 or type 2 diabetes, misdiagnosis or missed diagnosis is prone to occur.MODY7 was first discovered by Neve et al. in 2005 (2). In 2005, genetic screening was performed in two probands with a family history of early-onset type 2 diabetes mellitus. One patient was diagnosed with type 2 diabetes mellitus, and the other exhibited impaired glucose tolerance, implying that impaired pancreatic β-cell function and abnormal insulin secretion may be related to pathogenic gene variants. Distinct from other MODY subtypes, patients with MODY7 may present with dyslipidemia or fatty liver disease in addition to abnormal glucose metabolism. Moreover, MODY7 accounts for less than 1% of all MODY subtypes (2). *KLF11* is a pancreas-enriched transcription factor that has attracted considerable research interest due to its role as a negative regulator of exocrine cell growth both *in vitro* and *in vivo* (3). *KLF11* gene defects may lead to early-onset or polygenic type 2 diabetes, making it a potential therapeutic target for MODY (4).

In the present study, we report a case harboring a *KLF11* missense variant (p.Arg29Gln, R29Q) associated with MODY7. This variant has been previously documented in the literature. Patients carrying the *KLF11* R29Q variant generally present with a mild clinical phenotype and do not require insulin therapy at the early stage. Furthermore, not all carriers progress to overt diabetes. However, previous studies have been limited to case descriptions without in-depth investigations into the pathogenic mechanism of this mutation.

In this study, Proband 1 presented with impaired islet function, retinopathy, and severe hyperlipidemia at an early disease stage, the phenotype closely resembles that of type 1 diabetes mellitus, whereas most affected individuals in previous studies displayed a type 2 diabetes-like phenotype.

Proband 1 is not an isolated case. In the present study, we further identified Proband 2 in clinical practice, who also carries the *KLF11* R29Q variant and exhibits marked pancreatic β-cell dysfunction and a tendency toward insulin dependence. The above clinical heterogeneity suggests that the phenotypic expression of this variant may exhibit significant individual differences and may be modulated by factors such as genetic background and metabolic environment.

Accordingly, this study systematically investigated the clinical characteristics and potential pathogenic mechanisms of the *KLF11* R29Q variant by combining clinical phenotypic analysis and molecular genetic testing, and verified its effects on molecular function through *in vitro* functional experiments.

## 2 Materials and methods

### 2.1 Proband and family clinical data

**Figure 1:**
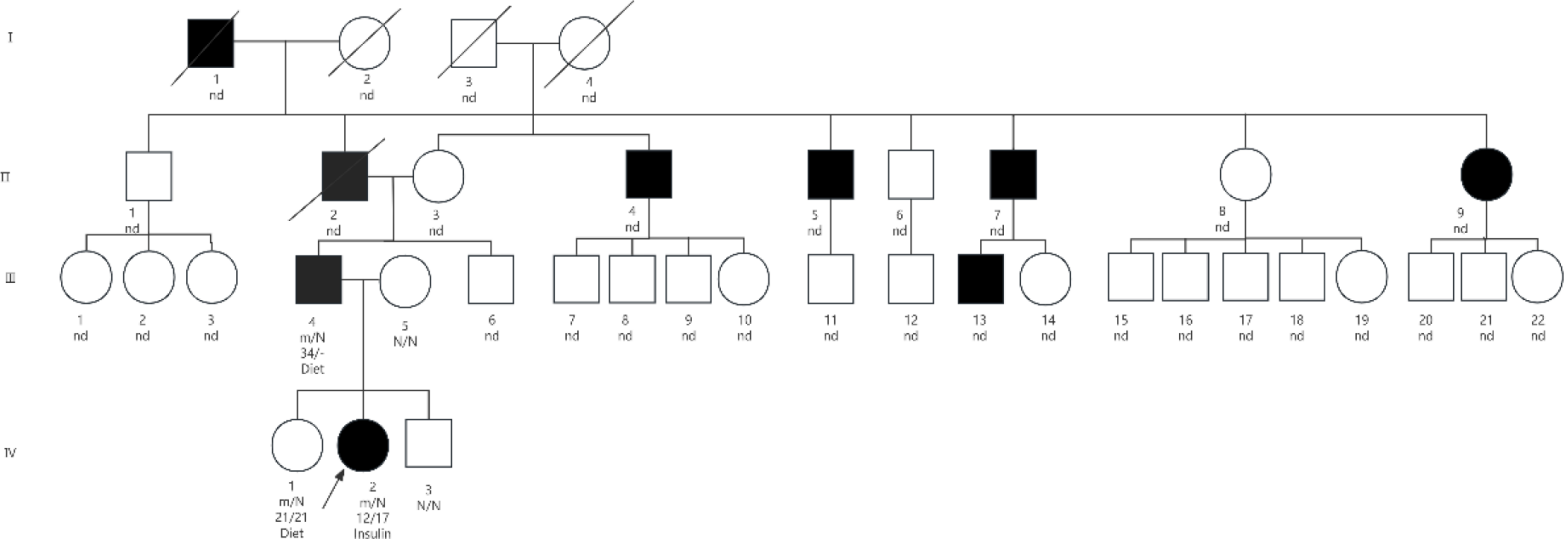
Pedigree of Proband 1 in this study. Black symbols represent diabetic patients, the arrow indicates the proband, and slashed symbols denote deceased individuals. The letters m and N represent the wild-type (WT) and p.Arg29Gln variant, respectively. The p.Arg29Gln variant was identified in individuals III-4, IV-1, and IV-2.

The proband 1 (The pedigree of the proband is shown in **Figure 1**, IV-2 is the proband) is female, born full-term by normal delivery (birth weight unknown). She was previously healthy, with no history of recurrent infections or diarrhea. At age 12, she developed polydipsia, polyuria and weight loss. Six months later, her vision significantly decreased. One year after symptom onset, she visited a local hospital due to pitting oedema in both lower limbs. Her blood glucose was 30.10 mmol/L, HbA1c was 17.7%, and serum lipid profiles were markedly elevated, with several parameters exceeding the upper limit of the laboratory’s analytical measurement range. Upon transfer to our hospital, urinalysis showed ketones 3+ and glucose 4 +. Arterial blood gas analysis showed pH 7.26, bicarbonate 7.4 mmol/L, and glucose 35.2 mmol/L. All five insulin autoantibodies were negative. Owing to severe lipemia, reliable measurements of biochemical parameters—including electrolytes, liver and renal function, myocardial enzymes, and serum lipids—could not be obtained due to assay interference. She received intravenous insulin for glycemic control and ketoacidosis management. Following correction of acidosis, insulin pump therapy was initiated to regulate blood glucose. During hospitalization, oral hypoglycemic agents were added but were ineffective. During subsequent hospitalization, insulin therapy was maintained. After discharge, she did not regularly monitor blood glucose or use insulin. At age 17, she was readmitted due to a finger infection. Poor glycemic control and severe infection led to finger amputation. Since then, insulin pump therapy has been continuously administered. At first visit, height was 143 cm, weight 31 kg, BMI 15.1 kg/m². At her last visit at age 18, height was 160 cm, weight 48 kg, BMI 18.75 kg/m². All five insulin autoantibodies (GAD, IA-2, IAA, ICA, ZnT8) were negative. When her blood glucose stabilized, an oral glucose tolerance test (OGTT) with insulin and C-peptide release tests was performed to assess islet function (**Table 1**).

**Table 1:** Blood glucose, insulin and C-peptide levels of Proband 1.

Time	Blood glucose mmol/L	Insulin uU/ml	C-Peptide ng/ml
0min	6.6	11.70	<0.01
120min	6.6	26.90	<0.01

The proband 1’s father (III-4) was diagnosed with diabetes at age 37. He took metformin orally but had poor glycemic control (fasting blood glucose >10 mmol/L) with relative insulin deficiency. He also had multiple xanthomas, indicating abnormal fat distribution. After switching to metformin combined with gliquidone, his blood glucose improved. The proband 1’s sister (IV-1) had a glucose level of 6.6 mmol/L and uric acid of 493 μmol/L. HbA1c, insulin antibodies, and thyroid function were normal. She had no clinical symptoms of diabetes and was in a prediabetic state, with possible future progression to diabetes. She was advised on diet and exercise modifications and regular blood glucose monitoring (**Table 2**). The proband 1’s great-grandfather (I-1), grandfather (II-2), three of the grandfather’s brothers (II-4, II-5, II-7), and one of the grandfather’s sisters (II-9) all had diabetes. The grandfather and his brothers were diagnosed between ages 45-50, had poor glycemic control with oral medication. Their BMI did not meet obesity criteria. The grandfather also had hyperlipidemia.

**Table 2:** Clinical characteristics of the Proband 1, father, and sister.

Clinical characteristics	Proband 1	Her father	Her sister
At the time of diagnosis			
Age (years)	12	37	–
Height (cm)	143	–	–
Weight (kg)	31	–	–
BMI (kg/m^2^)	15.1	–	–
DKA	Yes	–	–
Blood glucose(mmol/L)	30.1	–	6.6
HbA1c(NGSP,%)	17.7		5.8
The most recent time			
Age (years)	17	–	–
Height (cm)	160	–	–
Weight (kg)	53	–	–
BMI (kg/m^2^)	20.7	–	–
HbA1c(NGSP,%)	10.2	–	–
Fasting serum C-peptide (ng/mL)	<0.01	–	–
Fasting serum insulin (μU/mL)	–	2.87(mIU/L)	–
Treatment plan	Insulin pump combined with metformin	Metformin combined with glimepiride	Diet combined with exercise

Proband 2 was a female patient who presented with polydipsia, polyuria, and weight loss at the age of 8 years. She was admitted to a local hospital due to significant weight loss, where her fasting blood glucose was 20 mmol/L, and urinalysis showed 3+ proteinuria. Glycemic control was achieved after initiation of insulin aspart therapy. At the age of 9 years, she visited our outpatient clinic, and subsequent genetic testing identified the *KLF11* R29Q variant, which was inherited from her mother. The treatment regimen was adjusted to insulin pump therapy; however, hypoglycemia occurred during treatment. The insulin pump was therefore discontinued, and oral acarbose tablets were administered, resulting in satisfactory glycemic control. She was later admitted to our hospital due to poor glycemic control and slow growth. Insulin pump therapy was initiated for glycemic control, and recombinant human growth hormone was prescribed to improve her height. After discharge, the regimen was adjusted to subcutaneous injection of insulin aspart combined with insulin glargine. At the age of 13 years, she was readmitted to our hospital due to poor glycemic control. Laboratory examinations revealed glycated hemoglobin (HbA1c) of 11.4%, spot urine protein of 74.51 mg/g (later rechecked to be <12.3 mg/g), and trace ketonuria on urinalysis. Glutamic acid decarboxylase antibody was positive, while other biochemical parameters showed no obvious abnormalities. Autonomic function testing indicated autonomic neuropathy, and quantitative sensory testing suggested peripheral neuropathy. The hypoglycemic regimen was adjusted to insulin aspart, insulin glargine, and metformin. After her blood glucose gradually stabilized, an oral glucose tolerance test (OGTT) and C-peptide release test were performed to evaluate islet function (**Table 3**). Her parents were healthy, with no family history of diabetes. Her mother declined family segregation analysis; therefore, a pedigree chart could not be constructed. At the first visit, her height was 133.8 cm, weight 28 kg, and BMI 15.6 kg/m². At her most recent follow-up visit at the age of 14 years, her height was 156 cm, weight 45 kg, and BMI 18.49 kg/m².

**Table 3:** Blood glucose and C-peptide levels of Proband 2.

Time	0min	30min	60min	120min	180min
Blood glucose mmol/L	7.6	7.1	10.8	13.9	21.3
C-Peptide ng/ml	0.07	0.05	0.09	0.14	0.23

All procedures involving human participants complied with institutional and/or national ethical standards, the 1964 Helsinki Declaration and its later amendments, or comparable ethical standards. This study was approved by the Ethics Committee of The First Affiliated Hospital of Zhengzhou University (Approval No: 2025-KY-0605-003). All participants voluntarily signed informed consent forms, which were reviewed and approved by the Ethics Committee.

### 2.2 Mutation analysis

Peripheral blood samples were collected from Proband 1, her parents, elder sister and younger brother. High-throughput sequencing (NGS) was performed in Proband 1, and Sanger sequencing was used for validation in her parents, elder sister and younger brother. Whole-genome molecular genetic testing was conducted in Proband 1 and her elder sister. NGS was performed in Proband 2, and Sanger sequencing was used for validation in her parents.

### 2.3 Protein structure analysis of the *KLF11* variant

Expasy’s ProtParam tool was used to determine the physicochemical properties of wild-type and mutant *KLF11* proteins. The PSIPRED database was used to analyze secondary structure changes. ELM was used for short linear motif analysis. NetPhos-3.1 was used for phosphorylation analysis. SWISS-MODEL and PyMOL 2.0 were used for modeling and visualization. Missense3D was used to predict tertiary structure effects. AmberTools 20 in ChimeraX was used to analyze protein surface electrostatic potential. DUET, MUpro, DynaMut2, SAAFEC-SEQ, and I-Mutant2.0 SEQ were used to analyze the impact of the R29 mutation on *KLF11* protein stability.

### 2.4 Plasmid information

The vector pcDNA3.1-3xFLAG-C encoding the insulin promoter sequence was synthesized by Shanghai Yazai Biotechnology Co., Ltd. The extracted plasmids were transfected into MIN6 cells and divided into three groups: Vector plasmid, *KLF11*-WT plasmid, and *KLF11*-mut plasmid.

### 2.5 Western blotting analysis

MIN6 cells were transfected with plasmids encoding R29Q-*KLF11* and WT-*KLF11*. Cells were harvested 48 hours post-transfection for SDS-PAGE. Western blotting was performed using mouse monoclonal anti-FLAG-Tag primary antibody (Proteintech, Cat: 66008-4-Ig, 1:10000) and horseradish peroxidase (HRP)-conjugated horse anti-mouse IgG polyclonal secondary antibody (Cell Signaling Technology, Cat: #7076, 1:5000) or HRP-conjugated goat anti-rabbit IgG polyclonal secondary antibody (Cell Signaling Technology, Cat: #7074, 1:5000).

### 2.6 Luciferase reporter assay

MIN6 cells were seeded in 96-well plates (10,000 cells/well, 200 μL medium). After 24 hours, cells were transfected with pcDNA3.1-3xFLAG, pcDNA3.1-WT-*KLF11*, or pcDNA3.1-R29Q using Lipofectamine™ 2000 (Invitrogen, Cat: 11668027). After 48 hours post-transfection, dual-luciferase assays were performed using a kit (Promega, Cat: E1910).

### 2.7 Insulin secretion and insulin content assay

MIN6 cells were co-transfected with plasmids encoding R29Q-*KLF11* and WT-*KLF11* to investigate insulin secretion. After 48 hours post-transfection, cells were incubated in HEPES-balanced Krebs-Ringer bicarbonate buffer containing 0.5% BSA and different glucose concentrations. The medium was collected, and immunoreactive insulin was measured by ELISA (using mouse insulin as standard). Cells were dissolved in 200 μL of 1 mol/L NaOH per well for intracellular protein content measurement using an ELISA kit (Crystal Chem, 90080). Acidic ethanol (1 mL) was added to each well, incubated at 4 °C for 4 hours, and extracts were collected, diluted, and detected by ELISA to measure cellular insulin content within the cells.

### 2.8 Real-time PCR

Total RNA was isolated from MIN6 cells using Trizol reagent (BiyunTian, R0016). cDNA was synthesized using the TransScript All-in-One SuperMix for qPCR kit (Quanshijin, AT341). Real-time PCR was performed using a LightCycler 480. Primer sequences: Ins1 forward primer: CACTTCCTACCCCTGCTGG; Ins1 reverse primer: ACCACAAAGATGCTGTTTGACA;Actin forward prime: GTGACGTTGACATCCGTAAAGA;Actin reverse primer: GCCGGACTCATCGTACTCC. Actin served as the internal control.

### 2.9 Data analysis

Data analysis was performed using SPSS software (version 25.0).Differences between treatment groups were mainly analyzed using one-way analysis of variance (ANOVA) and t-test. ANOVA was used for comparisons among multiple groups, whereas the t-test was applied for comparisons between two groups. A significance level of *P* < 0.05 was used, indicating that a *P*-value below this threshold represents a statistically significant difference between the experimental and control groups.

## 3 Results

### 3.1 Clinical presentation

Proband 1 was first diagnosed with diabetes at the age of 13 years and received immediate insulin therapy, making it particularly important to determine the type of diabetes in this patient. Insulin treatment was continued outside the hospital; however, blood glucose fluctuated markedly due to external environmental factors (academic pressure, parental divorce, etc.) and emotional influences. At the age of 17 years, she was admitted to our hospital due to a finger infection. Subsequently, she was treated with an insulin pump combined with the hypoglycemic agent metformin, resulting in satisfactory glycemic control.

Proband 2 presented with the classic “three polys and one weight loss” symptoms of diabetes and initiated insulin therapy. She was subsequently admitted to our hospital multiple times due to poor glycemic control. Hypoglycemia occurred after switching to insulin pump therapy during treatment. In subsequent management, she was continuously treated with insulin aspart, insulin glargine combined with metformin. Oral glucose tolerance test (OGTT) and C-peptide release test indicated severely impaired islet function, accompanied by multiple diabetic complications including autonomic neuropathy and peripheral neuropathy.

### 3.2 Protein structure analysis of the *KLF11* R29Q variant

*KLF11* R29Q mutation changed the local amino acid structure from Coil to Helix, and the classic MAPK docking motif structure disappeared. The mutation may cause changes in secondary structure and local protein structure, potentially affecting MAPK kinase recognition and phosphorylation.

**Figure 2:**
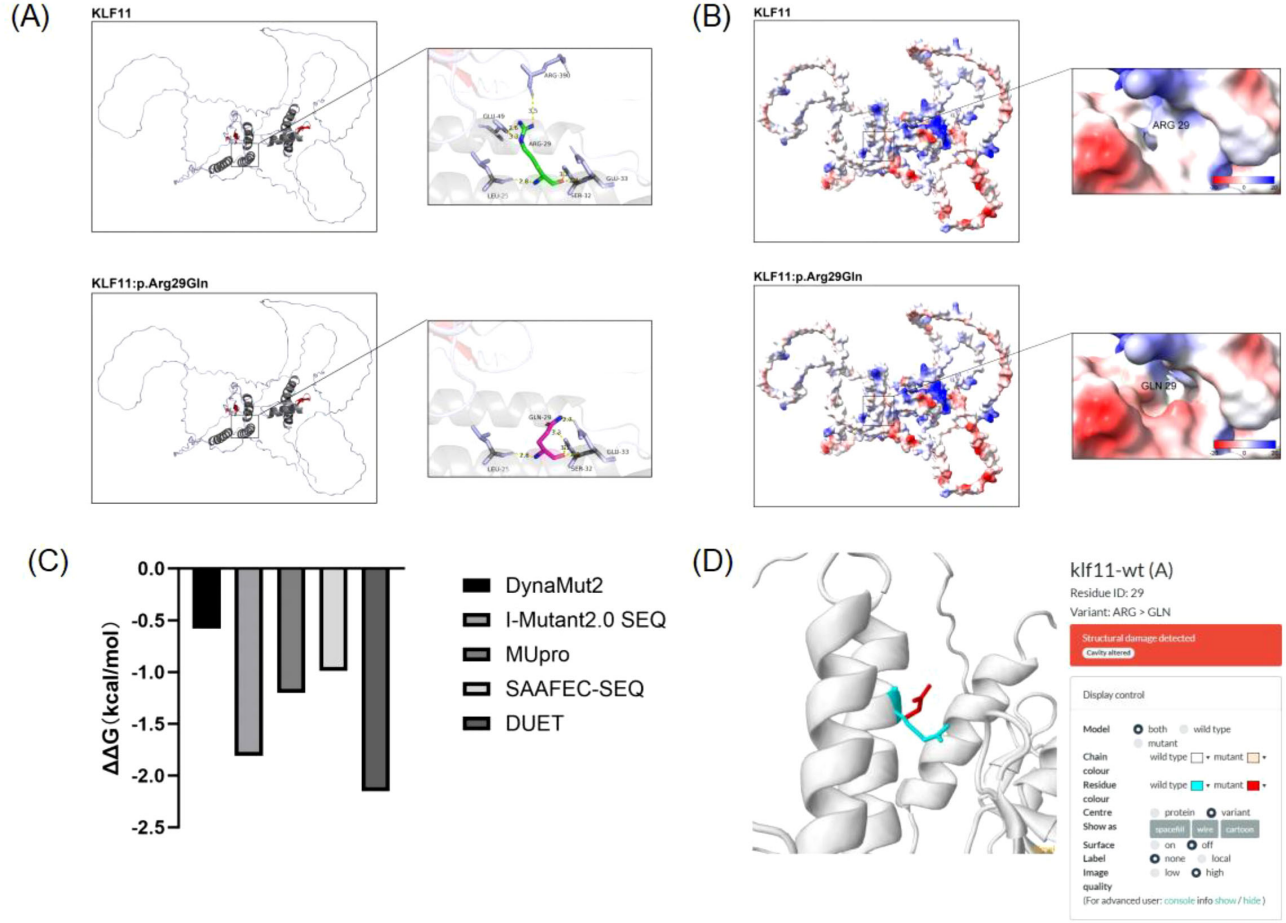
Protein structure predictions for *KLF11* (WT and R29Q). **(A)** Effect of *KLF11* R29Q mutation on tertiary structure: After mutation, the distance of the hydrogen bond between backbone GLN29 and SER32 changes, suggesting the mutation affects the backbone structure of *KLF11*. **(B)** Effect of *KLF11* R29Q mutation on protein surface electrostatic potential: The surface electrostatic potential at position 29 shifted from between positive and neutral towards between neutral and negative after mutation. The cavity morphology also changed, and its volume increased, suggesting the mutation affects the molecular surface electrostatic potential of *KLF11*. **(C)** Prediction of the effect of *KLF11* R29Q mutation on protein stability: Negative values indicate decreased protein stability, suggesting this mutation affects protein stability. **(D)** Prediction of the damaging effect of *KLF11* R29Q mutation on tertiary structure: The mutation has a damaging effect on the tertiary structure of *KLF11*.

Three-dimensional modeling demonstrated that the *KLF11* R29Q variant altered backbone and side-chain hydrogen bonds, which may affect the overall structural stability of the KLF11 protein. In particular, changes in backbone hydrogen bonds may influence local protein folding, while redistribution of side-chain hydrogen bonds may alter the three-dimensional conformation of the protein (**Figure 2A**). Furthermore, the electrostatic potential at position 29 shifted from between electropositive and neutral toward between neutral and electronegative after mutation, accompanied by an altered cavity morphology and increased cavity volume, suggesting that the mutation affects the electrostatic potential on the molecular surface of KLF11 (**Figure 2B**). The impact of the p.Arg29Gln mutation on KLF11 protein stability was analyzed using DUET, MUpro, DynaMut2, SAAFEC-SEQ, and I-Mutant2.0 SEQ, which indicated that this mutation may impair protein stability (**Figure 2C**). Missense3D prediction revealed that the major alteration induced by the *KLF11* R29Q mutation was Cavity altered (**Figure 2D**).

### 3.3 Functional characteristics of the *KLF11* R29Q variant

**Figure 3:**
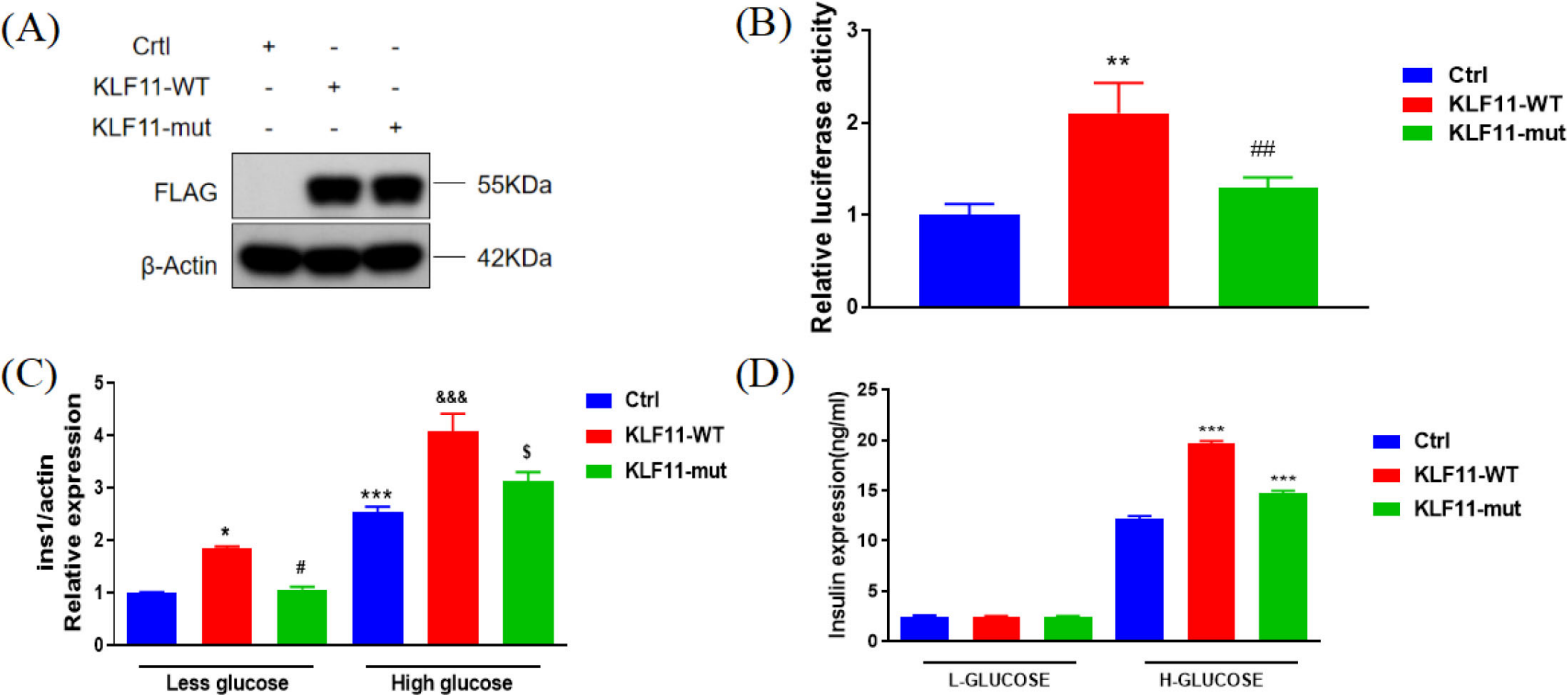
Functional analysis of *KLF11* R29Q mutation. **(A)***KLF11* (Wild-type *vs*. R29Q) protein expression. Protein levels were detected by western blotting using cell lysates from *KLF11*-expressing cells. **(B)** Luciferase assay in MIN6 cells transfected with different *KLF11* expression vectors (Wild-type *vs*. R29Q). In MIN6 cells treated with High Glucose, ** *vs* Ctrl p<0.01, ## *vs KLF11*-WT p<0.01. **(C)** Ins1 mRNA expression levels in MIN6 cells measured by qRT-PCR after 48-hour stimulation with 5.5 mmol/L glucose (Low) or 33.3 mmol/L glucose (High). In MIN6 cells, * *vs* Ctrl-Low glucose p<0.05, *** *vs* Ctrl-Low glucose p<0.001, ^#^*vs KLF11*-WT-Low glucose p<0.05; In MIN6 cells, ^&&&^*vs* Ctrl-High glucose p<0.001, ^$^*vs KLF11*-WT-High glucose p<0.05. **(D)** Insulin secretion levels in MIN6 cells measured by ELISA after glucose stimulation (5.5 mmol/L Low or 33.3 mmol/L High, 48 hours). In MIN6 cells, *** *vs* H-Glucose-Ctrl p<0.001.

Western blot analysis showed that the protein expression level of R29Q-*KLF11* in MIN6 cells was comparable to that of WT-*KLF11*, indicating that the *KLF11* R29Q gene variant does not affect protein expression levels (**Figure 3A**). Luciferase reporter assays showed that the insulin promoter activity induced by the *KLF11* R29Q variant was lower than that induced by WT-*KLF11*, indicating that this variant plays a role in the transcriptional regulation of the insulin gene (**Figure 3B**). To further investigate the impact of this variant on β-cell function, we overexpressed WT and *KLF11* R29Q variants in MIN6 cells (using pcDNA3.1 plasmid as a negative control for both *KLF11*-WT and *KLF11*-mut plasmids). We found that the *KLF11* R29Q variant reduced insulin transcription (**Figure 3C**) and decreased insulin secretion (**Figure 3D**), even under high glucose stimulation.

## 4 Discussion

In this study, we report a heterozygous *KLF11* R29Q mutation in two diabetic patients and their family members. This mutation has been documented previously (2) and was classified as a variant of uncertain clinical significance. However, combined with the typical clinical manifestations of the two patients in this study, we diagnosed them with MODY7.In the family of Proband 1, diabetes was present in four generations. However, her marked pancreatic β-cell dysfunction was evident early in the disease course. She also presented with severe hyperlipidemia, with a much higher degree of dyslipidemia than previously reported in the literature. The father of Proband 1 was diagnosed with diabetes at 37 years of age and presented with abnormal fat distribution, such as multiple xanthomas on the eyelid. Her elder sister was in a prediabetic state and did not require insulin therapy. At diagnosis, Proband 2 exhibited marked pancreatic β-cell dysfunction with negative insulin antibodies, manifesting a clinical phenotype consistent with insulin-dependent diabetes. Although his mother harbored the *KLF11* p.Arg29Gln (R29Q) variant, she maintained normal glycemic levels without any diabetes-related clinical manifestations. According to the diagnostic criteria for MODY, including autosomal dominant inheritance, a family history of diabetes spanning more than three generations, onset before 25 years of age, and negative insulin antibodies (5, 6), both patients were diagnosed with MODY7.However, compared with the characteristics of previously reported MODY7 cases, the two patients in the present study differed distinctly: they presented with marked insulin deficiency early in the disease course and developed multiple diabetes-related complications. To investigate the marked phenotypic differences between these patients and their relatives, we performed functional studies to clarify whether this variant contributes to the abnormal phenotypes observed in these patients.

*KLF11* is a zinc finger transcription factor, a member of the SP/KLF family of DNA-binding regulatory protein (3). Its functions include tissue-specific regulation of pancreatic acinar SMAD7, SOD2, catalase 1, and insulin gene expression in islet cells (4, 7). *KLF11* is the causative gene for MODY7. Research shows that *KLF11* binds directly to specific sequences in the insulin promoter, exerting a transcriptional activation effect, increasing insulin expression, and participating in maintaining pancreatic β-cell function and pancreatic development (7–9). *In vitro* experiments confirm that *KLF11* gene mutations block its binding to the insulin gene promoter, thereby inhibiting transcriptional activation and downregulating insulin expression. In systemic *KLF11* knockout mouse models, pancreatic islet number and volume significantly decreased, with obvious islet developmental abnormalities. Plasma insulin levels and insulin gene mRNA expression were significantly lower, indicating severely impaired insulin synthesis function in *KLF11* knockout mice (10).

**Figure 4:**
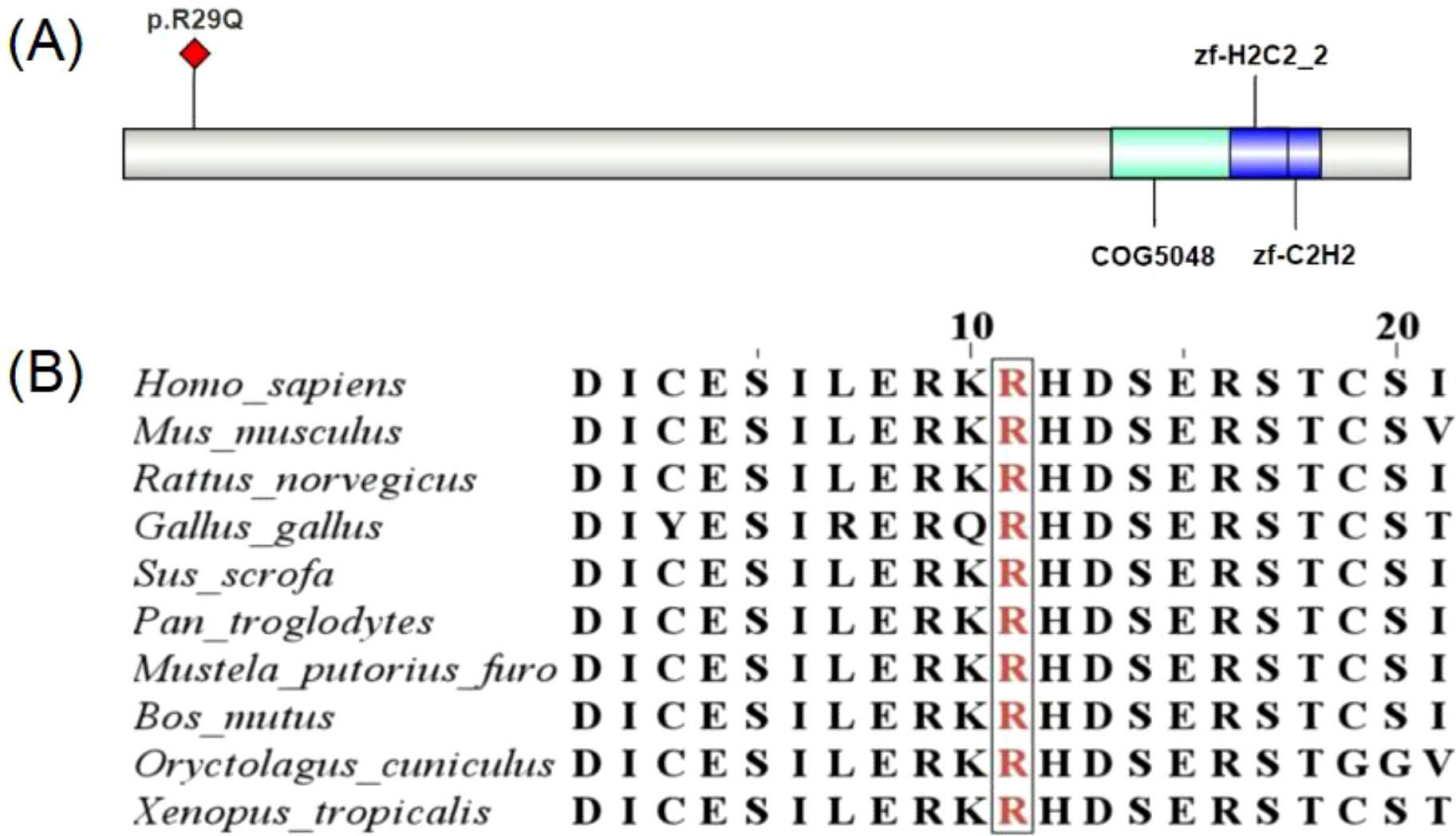
**(A)***KLF11* domains, NCBI prediction shows the R29 mutation site is not located within a known functional domain. **(B)** Cross-species conservation of *KLF11* R29Q mutation.

Our results indicated that multiple amino acid sequence alignment showed the KLF11 R29Q variant to be located at a highly conserved site (see **Figure 4**), implying its functional importance throughout evolution. Western blot analysis showed no significant difference in protein expression levels between cells transfected with *KLF11*−WT and *KLF11* R29Q.Subsequently, we performed *in vitro* assays using vectors containing wild−type and mutant *KLF11*. Luciferase reporter assays indicated that insulin promoter activity induced by the *KLF11* R29Q variant was significantly lower than that of *KLF11*−WT, suggesting that the mutation impaired the regulatory activity of *KLF11* on the insulin promoter. Further qRT−PCR and glucose−stimulated insulin secretion (GSIS) assays demonstrated that the *KLF11* R29Q variant reduced insulin transcription and decreased insulin secretion even under high glucose stimulation. Taken together, these *in vitro* experiments confirmed that the *KLF11* R29Q mutation indeed impairs islet function in the patients.

According to the diabetes classification guidelines, type 1B diabetes is defined as a form of diabetes caused by insulin deficiency due to non-autoimmune β-cell destruction (11). Previous studies have shown that a small proportion of patients clinically diagnosed with type 1B diabetes carry pathogenic gene mutations responsible for MODY, and that *KLF11* mutations can also lead to incompletely penetrant childhood type 1B diabetes (12–15). In a Japanese family, three patients presented with poor islet function and required insulin therapy at the early disease stage, all exhibiting a type 1 diabetes-like phenotype, which is consistent with the present study, accumulating evidence has demonstrated that the poor islet function in patients may result from a dominant-negative effect of the *KLF11* gene mutation (15). The mutation was also detected in their unaffected maternal grandmother, indicating incomplete penetrance. In the present study, the elder sister of Proband 1 showed relatively preserved islet function, with only mildly elevated fasting glucose and a prediabetic state. The mother of Proband 2 had normal blood glucose and no diabetes-related clinical manifestations, showing obvious phenotypic differences from the probands. These findings suggest that *KLF11* gene mutations may exhibit incomplete penetrance and could be associated with epigenetic alterations (16).

Non-genetic factors, including environment, lifestyle and dietary habits, may act as potential modulators of phenotypic plasticity in monogenic diseases by influencing the expression of modifier genes (17). The patient in this study had adverse lifestyle habits, such as smoking, tattooing and staying up late. It cannot be excluded that environmental factors contributed to the striking phenotypic differences between the patient and her sister. However, studies have suggested that *KLF11* mutations may cause diabetes only when combined with certain other genetic or environmental factors (15).

Phenotypic heterogeneity is not only observed among patients with MODY7; many individuals harboring the same pathogenic variant still exhibit extensive heterogeneity in disease expression, suggesting that factors other than the causative gene contribute to disease risk (18). A polygenic background is considered a likely contributor, particularly in age-dependent monogenic diseases (19). The genetic architecture of MODY appears more complex than its traditional characterization as a purely monogenic disorder. Among clinically confirmed cases, common genetic variants explain approximately 24% of phenotypic variance. Pathogenic MODY variants drive early-onset disease, while the polygenic background modifies overall disease risk. The contribution of polygenicity is not constant but depends on the underlying pathogenic variant, with less disruptive variants exerting a greater impact on disease expression and clinical diagnosis (20, 21). Therefore, the marked phenotypic differences between the patients and their family members in the present study cannot exclude the influence of a polygenic background.

Most MODY7 patients require insulin therapy at initial diagnosis, although sulfonylureas are also recommended for treating MODY7 patients (22, 23). In this study, the patient received insulin therapy initially, but required high doses. Subsequent combination with a sulfonylurea allowed for insulin dose reduction, but insulin could not be completely discontinued. After developing an infection, the patient continued using an insulin pump combined with metformin, achieving acceptable glycemic control.

## 5 Conclusion

In this study, we demonstrated that the *KLF11* R29Q variant is involved in the pathogenesis of MODY7. This variant may affect the 3D structure of the *KLF11* protein molecule, impairs its insulin promoter regulatory activity, and damages insulin expression while inhibiting insulin secretion in pancreatic β-cells. Our study also has limitations. It did not explain the relationship between the altered *KLF11* function and disease severity, or whether external factors influence the *KLF11* variant. This still requires further research, such as iPSC and animal studies.

## Data Availability

The datasets presented in this study can be found in online repositories. The names of the repository/repositories and accession number(s) can be found in the article/supplementary material.
